# The PPI network analysis of mRNA expression profile of uterus from primary dysmenorrheal rats

**DOI:** 10.1038/s41598-017-18748-2

**Published:** 2018-01-10

**Authors:** Pei Fan, Qiao-Hui Lin, Ying Guo, Lan-Ling Zhao, He Ning, Meng-Ying Liu, Dong-Qing Wei

**Affiliations:** 1 College of Biological Engineering, Henan University of Technology, Zhengzhou, 450001 China; 2 College of Chemical Engineering and Environment, Henan University of Technology, Zhengzhou, 450001 China; 3 College of Life Science and Biotechnology and State Key Laboratory of Microbial Metabolism, Shanghai Jiao Tong University, Shanghai, 200240 China

## Abstract

To elucidate the mechanisms of molecular regulations underlying primary dysmenorrhea (PD), we used our previously published mRNA expression profile of uterus from PD syndrome rats to construct protein-protein interactions (PPI) network via STRING Interactome. Consequently, 34 subnetworks, including a “continent” (Subnetwork 1) and 33 “islands” (Subnetwork 2–34) were generated. The nodes, with relative expression ratios, were visualized in the PPI networks and their connections were identified. Through path and module exploring in the network, the bridges were found from pathways of cellular response to calcium ion, SMAD protein signal transduction, regulation of transcription from RNA polymerase II promoter in response to stress and muscle stretch that were significantly enriched by the up-regulated mRNAs, to the cascades of cAMP metabolic processes and positive regulation of cyclase activities by the down-regulated ones. This link is mainly dependent on *Fos/Jun - Vip* connection. Our data, for the first time, report the PPI network analysis of differentially expressed mRNAs in the uterus of PD syndrome rats, to give insight into screening drugs and find new therapeutic strategies to relieve PD.

## Introduction

Primary dysmenorrhea (PD), a frequently occurring gynaecological disease that poses burdens to the young women with the symptoms^1^, has been proven to be closely related to the abnormal contraction of uterine smooth muscle cell (USMC)^2^. The smooth muscle contraction and relaxation are critically dependent on multiple biological pathways. For instance, calcium ion is required for initiating smooth muscle contraction^3^. Furthermore, mitogen-activated protein (MAP) kinases, SMAD and NF-kappa B pathways play key roles in promoting the contractility^4–7^. On the other hand, cAMP is the second messenger that causes smooth muscle relaxation^8^. These essential pathways are coordinately maintaining the normal functions of smooth muscle. In case of the balance being disturbed, the abnormal contraction-relaxation of smooth muscle may be present and the relevant disease symptoms emerge thereof.

To disclose the regulatory mechanisms of PD in molecular level, we previously compared the mRNA expressive differences in the uteruses between normal and PD syndrome rats. The data showed that, in the uterus of PD syndrome rats, 267 and 415 mRNAs were up- and down-regulated, respectively. These differentially expressed genes (DEGs) were significantly enriched in a series of pathways in rat uterus that are associated with the USMC functionalities^9^. It revealed that some essential biological processes, such as cellular response to calcium ion and SMAD protein signaling transduction, were fortified in the uterus of PD syndrome rats. In contrast, some other functions, including cAMP metabolic process and monovalent inorganic cation homeostasis, were attenuated. These may cooperatively contribute to the enhanced USMC contraction that results in PD.

Within cells, proteins function integratively through protein-protein interactions (PPI), which is essential for almost all biochemical activities to achieve specific tasks in life^10,11^. PPI also endows a single protein with multiple functions^12,13^. Therefore, investigations on PPI methodologies and applications to disclosing mechanisms of biological processes draw increasing attention^11,14–16^. For deeply understanding the regulatory mechanisms in many diseases, PPI networks generated by analyzing DEGs in the diseases are well performed^13,17,18^. However, in the uterus of PD syndrome rats, PPI network analysis for DEGs still remains unknown. In this study, the 682 DEGs were analyzed to construct PPI network, combined with previous reported GO classification and enrichment, for the purpose of comprehensively unraveling the molecular regulatory mechanisms in PD.

## Results

### The PPI network of DEGs

**Figure 1:**
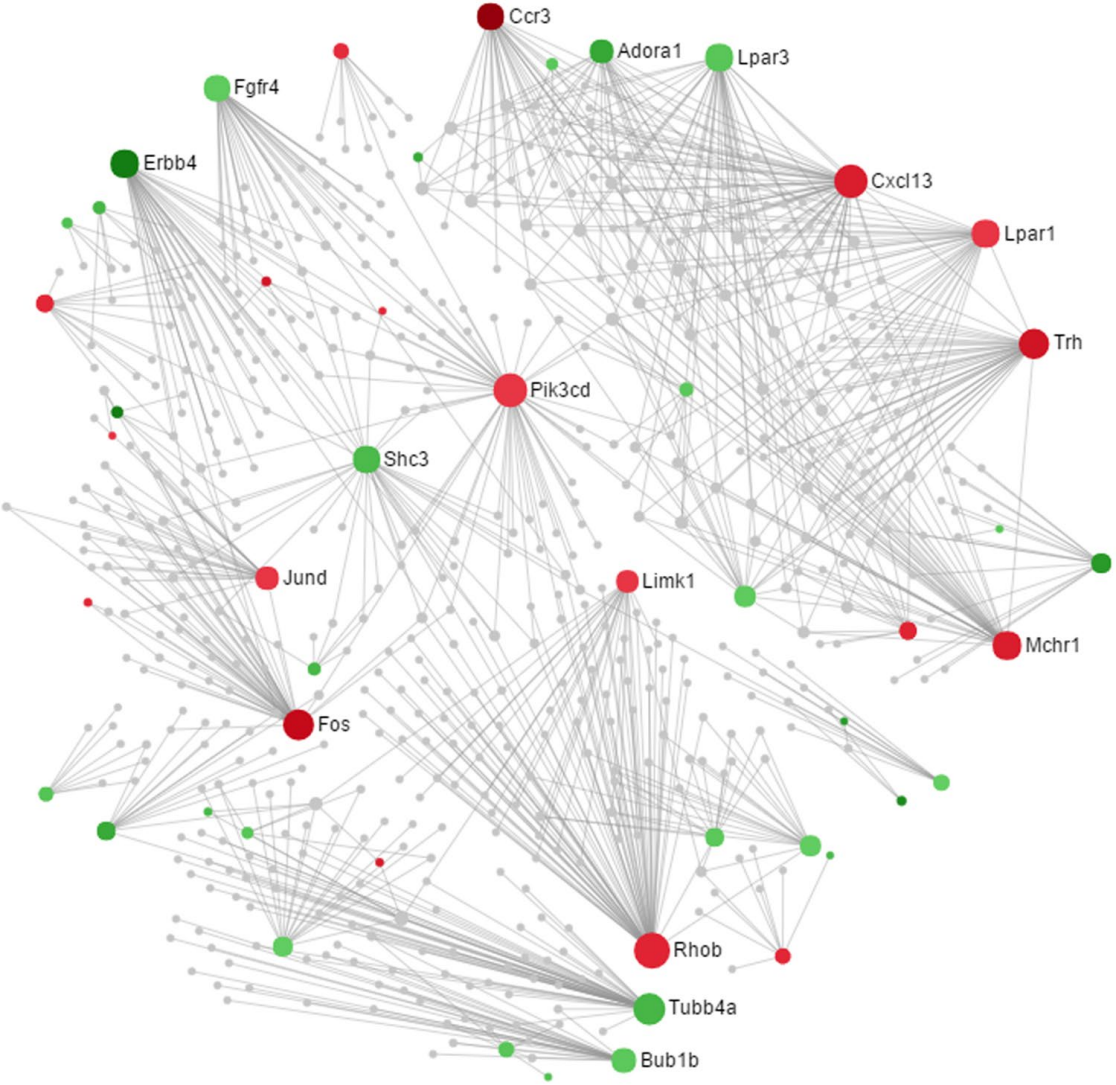
The PPI network of DEGs in the uterus of PD syndrome rats. The network displayed is “continent” (Subnetwork 1). The colors represent the expressions of nodes. Specifically, “red” and “green”indicate the nodes are up- and down-regulated, respectively. The grades of the colors represent the expression levels. The areas of the nodes indicate the degrees that the nodes connect to others. The nodes with gene names are the top 17 hub nodes in the PPI network.

**Figure 2:**
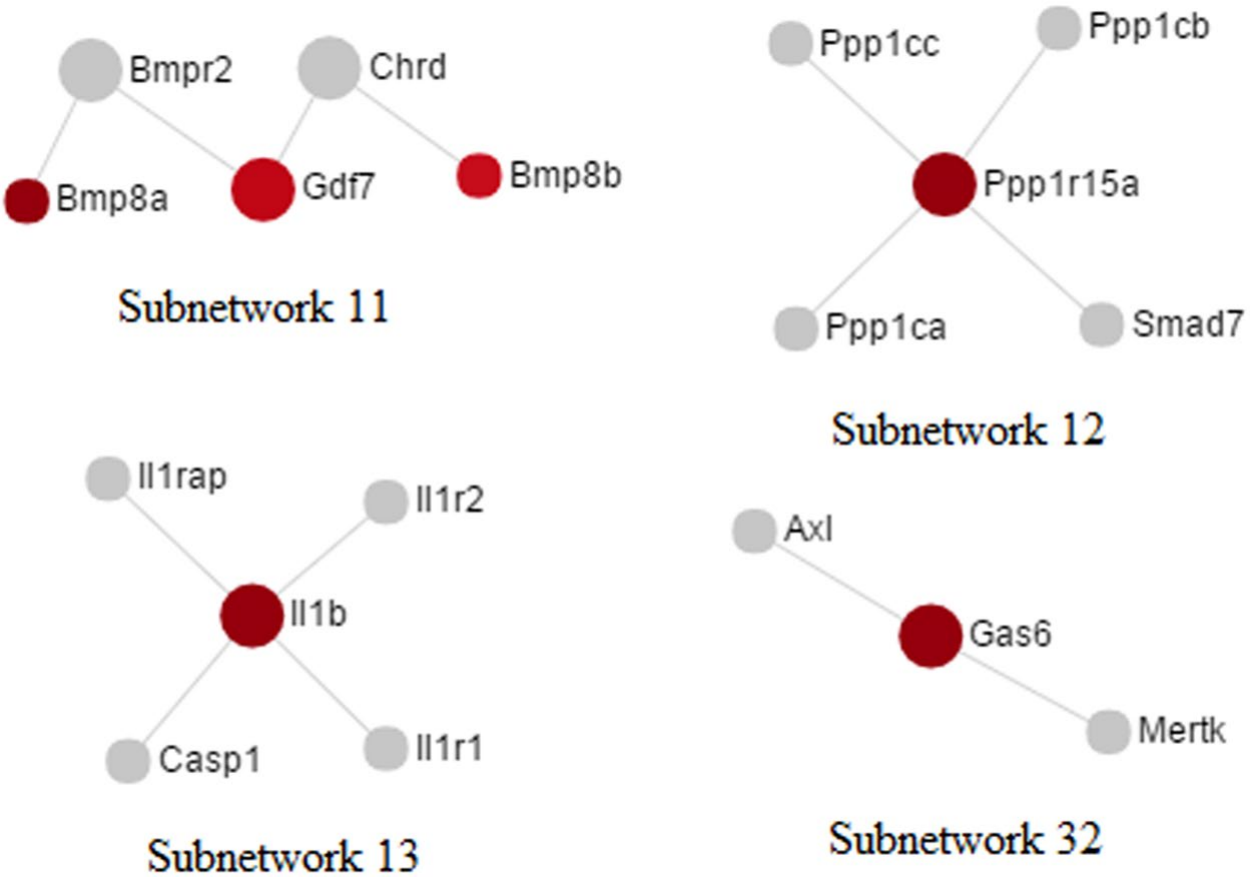
Subnetworks including up-regulated nodes implicated in the pathways that are relevant to USMC contraction and PD regulation. The nodes in red color indicate that they are up-regulated in the uterus of PD syndrome rats. The grade of color and the area of the node in the network represent the expression level and the degree of the node, respectively.

**Figure 3:**
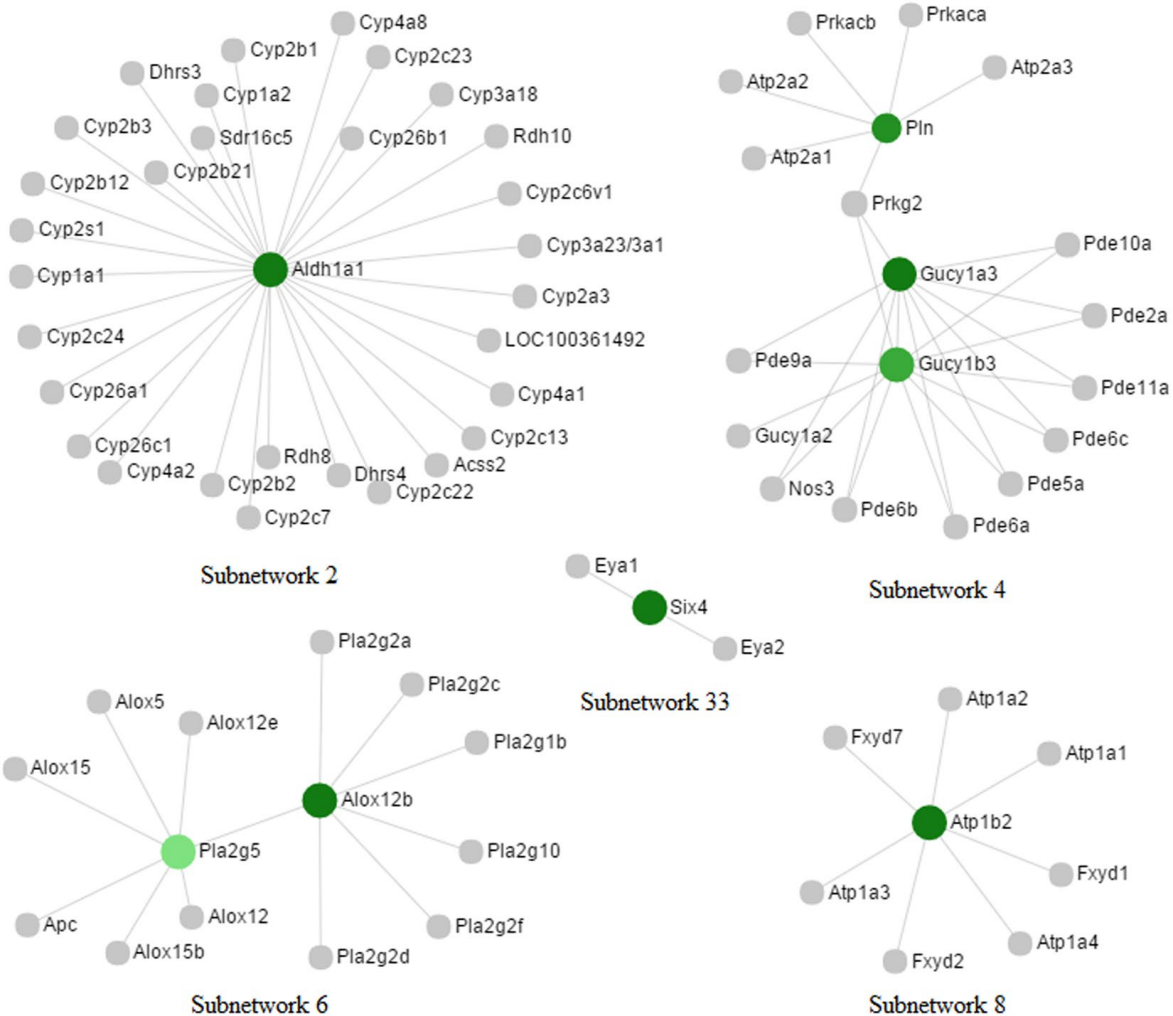
Subnetworks that contain down-regulated nodes implicated in the pathways. Nodes in green color are down-regulated in the uterus of PD syndrome rats. The grade of the color represents the expression level of the node while the area indicates the degree of the node in the network.

The total 682 DEGs in the uterus of PD syndrome rats were analyzed together to construct the PPI network. Consequently, 34 subnetworks, including a “continent” (Subnetwork 1) and 33 “islands” (Subnetwork 2–34), were generated. The visualized Subnetwork 1, shown in Fig. 1, contained 607 nodes, 848 edges and 49 seeds. The visualized Subnetwork 2–34 were demonstrated in Figs 2 and 3 and Supplemental Figs S1–5. The numbers of nodes, edges and seeds in all subnetworks were displayed in Supplemental Table S1. In the visualized networks, the expressions and the degrees of connections of the nodes were symbolized by their colors and areas, respectively.

### Hub nodes in the network

**Figure 4:**
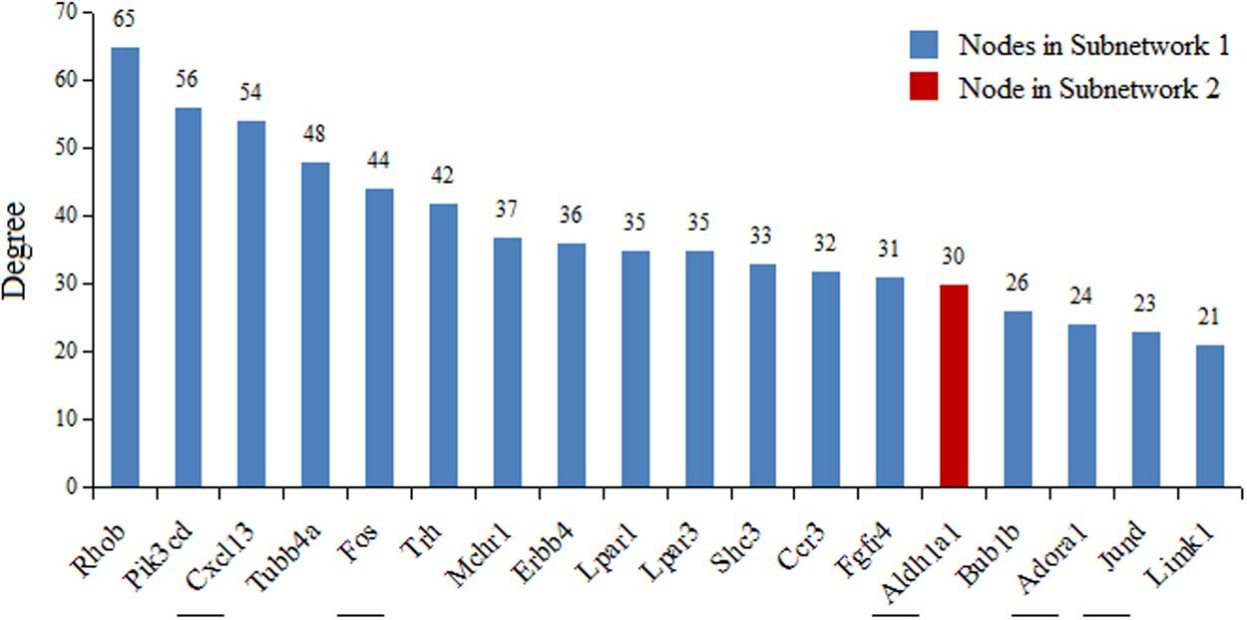
The hub nodes in the PPI network. The top 18 hub nodes with their degrees are displayed, in which genes with blue and red columns are in the Subnetwork 1 and Subnetwork 2, respectively. Genes with underlying bars are the DEGs implicated in the pathways shown in Supplemental Table S2.

**Figure 5:**
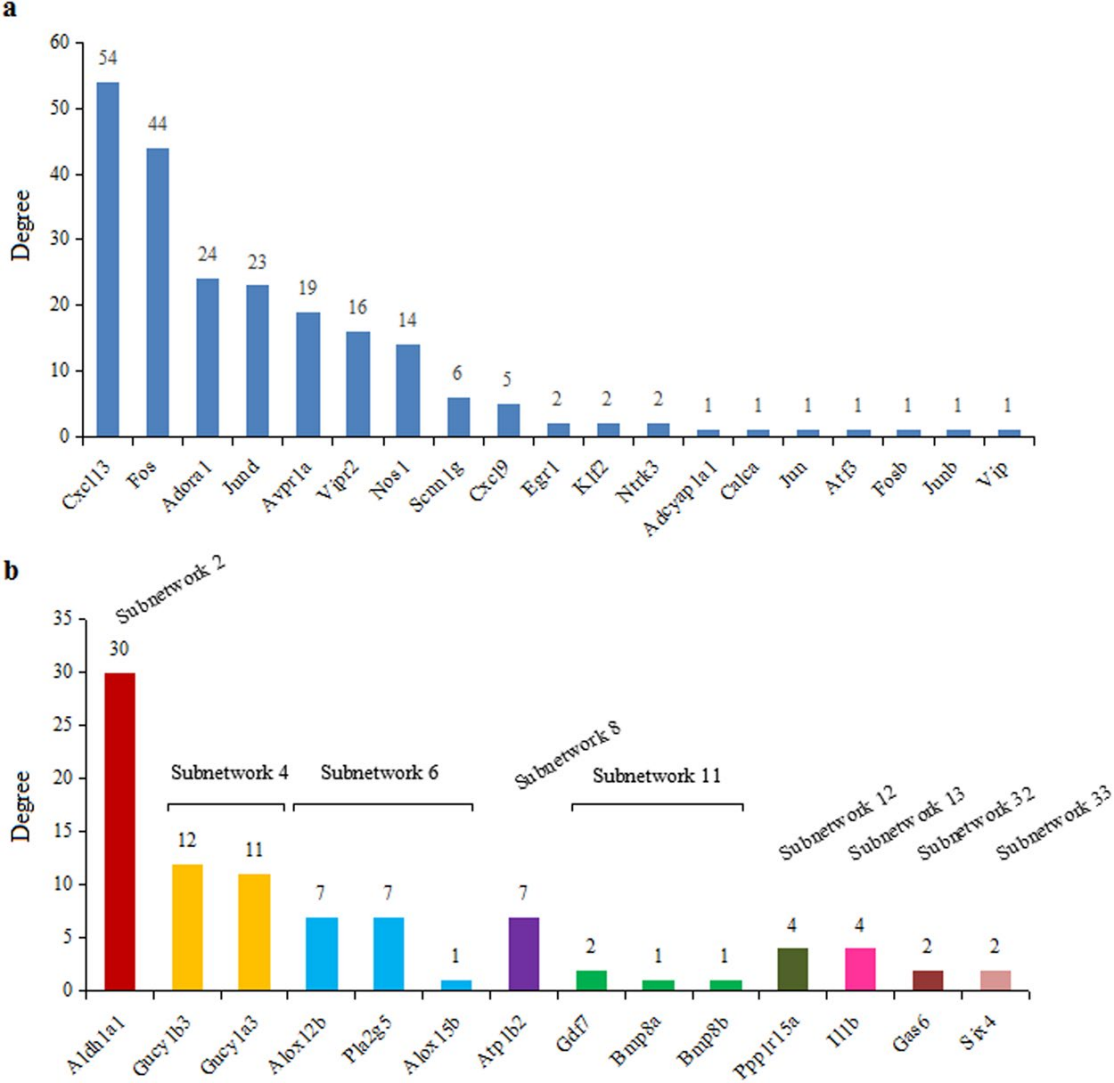
The degrees of nodes implicated in the significantly enriched pathways by DEGs in the PPI network. **(a)** shows the degrees of the nodes in Subnetwork 1; **(b)** demonstrates the degrees of the nodes in other subnetworks. The same color of the columns indicates that the genes are in the same subnetwork.

The top 18 hub nodes in the entire PPI network with their degrees were shown in Fig. 4. Particularly, 17 of them were included in Subnetwork 1 that were also highlighted in Fig. 1. Aldehyde dehydrogenase 1 family, member A1 (*Aldh1a1*), included in Subnetwork 2, was visualized in the network in Fig. 3. Furthermore, the nodes implicated in the significantly enriched pathways that are relevant to USMC contraction and PD regulation (Supplemental Table S2) were displayed with degrees in Fig. 5. To be specific, nodes included in the “continent” and the “islands” were shown in Fig. 5a and b, respectively. Additionally, these nodes were visualized in the subnetworks in Figs 2 and 3.

### Connections between functions in the network

**Figure 6:**
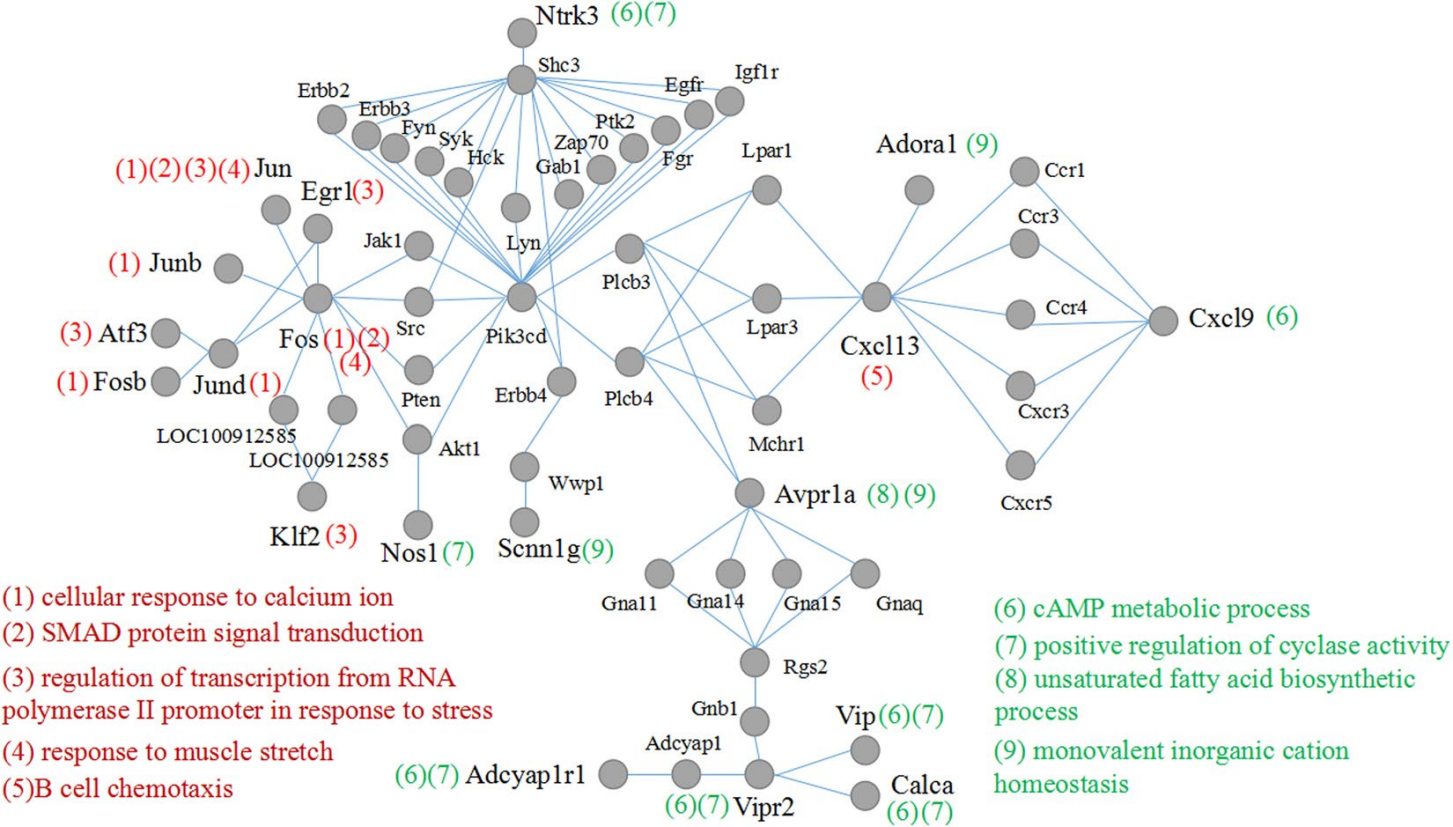
Connections between nodes implicated in the pathways in the PPI network. Pathways in red or green colors represent that they are significantly enriched by the up- or down-regulated mRNAs, respectively. The number in the bracket, representing the pathway, is adjacent to the node to indicate that the node is implicated in the pathway.

By virtue of path explorer, the connections of nodes that are in the specific biological pathways were redesigned in Fig. 6. As is shown, pathways of cellular response to calcium ion, SMAD protein signal transduction, regulation of transcription from RNA polymerase II promoter in response to stress, response to muscle stretch, and B cell chemotaxis that are significantly enriched by the up-regulated DEGs contained the nodes connecting the PPI network (Subnetwork 1); pathways of cAMP metabolic process, positive regulation of cyclase activity, unsaturated fatty acid biosynthetic process, and monovalent inorganic cation homeostasis that are significantly enriched by the down-regulated DEGs also had the connected nodes with the network (Subnetwork 1). Noticeably, These cascades were functionally linked to one another by certain nodes in the network.

### Modules in the network

**Figure 7:**
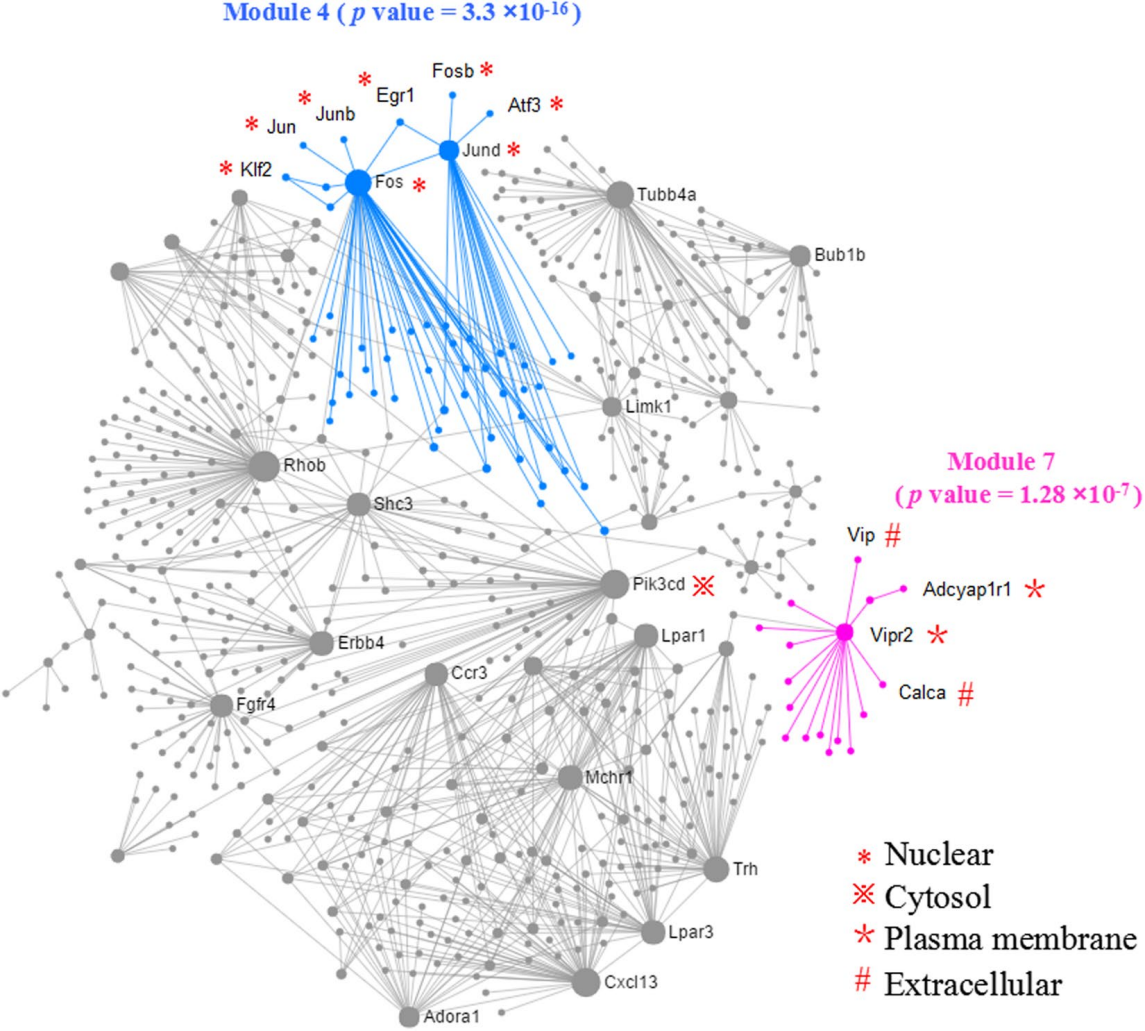
Module 4 & 7 in the PPI network. The modules in blue and pink colors are the Module 4 and Module 7, respectively. The areas of the nodes represent the degrees of the nodes that connect to others in the network. The special symbol with the node is shown to be the protein subcellular localization in the network.

The PPI network was composed of 19 modules shown in Supplemental Table S3 via module exploring function. Particularly, Module 4 and 7, with respective *p* values of 3.3 × 10^−16^ and 1.28 × 10^−7^, contained most of the nodes that are in the USMC contraction and PD related pathways. The two modules were demonstrated in different colors in Fig. 7. This may be indicative of the key roles of Module 4 and 7 in the PPI network in regulating PD.

### Protein-drug interactions

**Figure 8:**
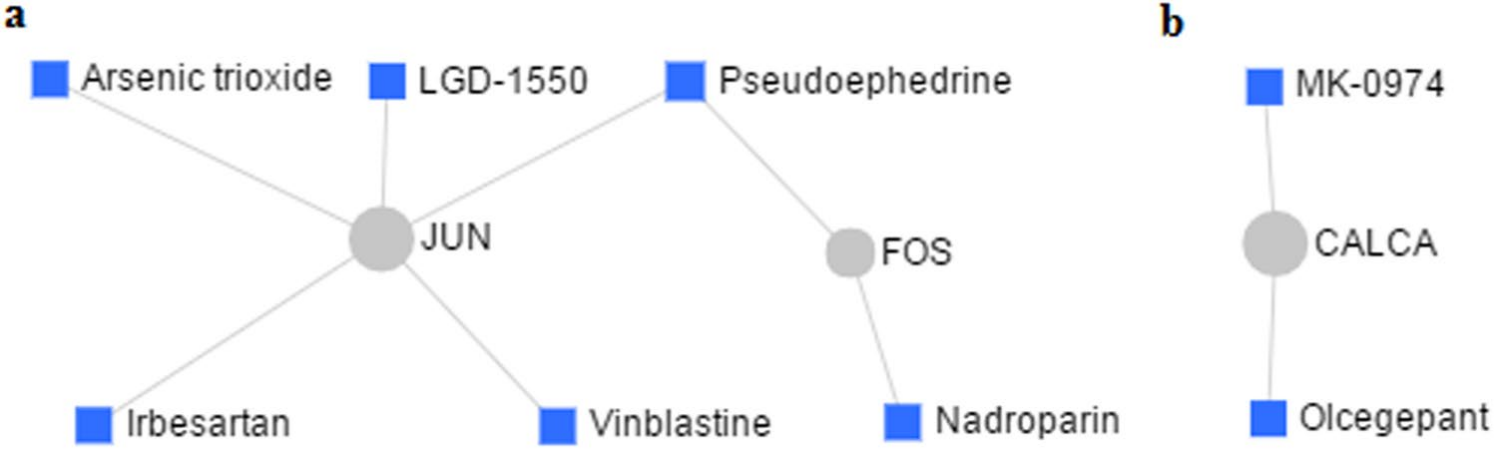
Protein-drug interactions network. (**a**) is the interactions between drugs and up-regulated nodes in the uterus of PD syndrome rats; (**b**) shows the interactions between drugs and down-regulated nodes. The area of the node represents the degree of interaction in the network.

The protein-drug interactions network was composed of two subnetworks. Subnetwork 1 contained 7 edges and 2 seeds; Subnetwork 2 included 2 edges and one seed. In Subnetwork 1, jun proto-oncogene (*Jun*) connected to the drugs of arsenic trioxide, irbesartan, vinblastine and LGD-1550 while FBJ osteosarcoma oncogene (*Fos*) connected to nadroparin. Noticeably, both *Jun* and *Fos*, the two up-regulated mRNAs in the uterus of PD syndrome rats, were linked by pseudoephedrine (Fig. 8a). In Subnetwork 2, calcitonin gene-related peptide alpha (*Calca*) that is down-regulated was connected to olcegepant and MK-0974 (Fig. 8b).

### Protein subcellular localization

The subcellular localization of proteins of all the DEGs in the uterus of PD syndrome rats was shown in Supplemental Table S4. The distributions and percentages of the proteins in subcellular levels were displayed in Supplemental Fig. S6. Particularly, nodes of *Fos*, jun D proto-oncogene (*Jund*), *Jun*, jun B proto-oncogene (*Junb*), FBJ osteosarcoma oncogene B (*Fosb*), early growth response 1 (*Egr1*), activating transcription factor 3 (*Atf3*) and Kruppel-like factor 2 (*Klf2*) in Module 4 in the PPI network were localized in nucleus. In Module 7, nodes of vasoactive intestinal peptide receptor 2 (*Vipr2*) and adenylate cyclase activating polypeptide 1 receptor 1 (*Adcyap1r1*) were localized in plamsa membrane while nodes of vasoactive intestinal peptide (*Vip*) and *Calca* were distributed extracellularly. Additionally, phosphatidylinositol-4,5-bisphosphate 3-kinase, catalytic subunit delta (*Pik3cd*), the bridge linking Module 4 & 7, was in the position of cytosol. This indicates that these genes may involve in PD regulation in a stepwise manner from nucleus to plasma membrane/extracellular position through cytosol. The subcelluar localization of these nodes were marked in Fig. 7.

## Discussion

PD has complicated regulatory mechanisms. Our previous study revealed that several essential pathways involving in smooth muscle contraction were significantly enriched by the DEGs in the uterus of PD syndrome rats^9^. Thereafter, in this study, PPI network analysis of the DEGs were performed to disclose the relations between the cascades and the genes. It can be inferred from the PPI network that the essential biological processes are functionally connected. By module exploring, the up-regulated nodes, including *Fos*, *Jun*, *Junb*, *Klf2*, *Egr1*, *Jund*, *Fosb* and *Atf3*, implicated in the pathways of cellular response to calcium ion, SMAD protein signal transduction, regulation of transcription from RNA polymerase II promoter in response to stress, and response to muscle stretch that are crucial to UMSC contraction, were clustered in the same module. This is an indication that these genes act together to promote the USMC contraction.

*Fos* family is composed of *Fos*, *Fosb*, FOS like 1, AP-1 transcription factor subunit (*Fosl1*) and FOS like 2, AP-1 transcription factor subunit (*Fosl2*)^19^. In our study, *Fos* and *Fosb* were up-regulated in the uterus of PD syndrome rats. *c-Fos*, the product of *Fos* with a transactivation domain at C-terminus, is up-regulated with the increase of contractile phenotype markers, including smooth muscle protein 22 alpha, alpha-actin, and calponin, in vascular smooth muscle cells (VSMCs) in response to cyclic strain^20^. This shows that *c-Fos* is associated with smooth muscle differentiation and contraction, indicating that *Fos* family may contribute to the USMC contraction and result in PD. In fact, evidences also show that *Fosb* is up-regulated in smooth muscle exposed to mechanical stimuli^21–23^. In addition, the members of *Jun* family, including *Jun*, *Junb* and *Jund*, were also up-regulated in the uterus of PD syndrome rats. *Fos* and *Jun* families form a series of dimeric complexes known as transcription factors of activator protein 1 (AP-1) family^24^. In *Jun* family, *Junb* has been shown to positively regulate basal- and TGFβ1-induced contraction of bladder smooth muscle cell^25^. Evidence also revealed that the *Junb* deficient mice were displayed the phenotype of decreased hypertension induced by DOCA-salt and attenuated contractile capacity of arterial smooth muscle cells^26^. Previous study showed that mRNA levels of *c-Fos*, *Fosb*, *c-Jun*, *Junb*, and *Jund* are all increased in rat aorta after vascular injury^27^. As the *Jun* family member, *Jund* has the similar expression trend as *c-Jun* and *Junb* in VSMC and USMC, which indicates that *Jund* may be functionally coordinated with other members of *Jun* family in smooth muscle cells.

AP-1 family plays essential roles in cell proliferation and survival^28^, which critically involves in MAP kinases pathways in various cellular types^29–31^. Previous data revealed that the extracellular signal-regulated kinases (ERK) and the c-Jun NH2-terminal kinases (JNK), two essential members of MAP kinases, can be activated in VSMCs from the acutely induced hypertensive rats, with the up-regulated gene expression of *c-Fos* and *c-Jun*, as well as the enhanced AP-1 DNA-binding activity^32^. It was also proven that the *c-Fos* mRNA and AP-1 DNA-binding activity are elevated by the activation of ERK1/2 in VSMCs^33^. Therefore, the up-regulation of *Fos*/*Jun* family members in the uterus of PD syndrome rats may contribute to USMC contraction via the association with MAP kinases signaling pathways.

VIP, shown to be down-regulated in the uterus of PD syndrome rats in our data, has effects on airway smooth muscle relaxation, bronchodilation and vasodilation^34^. It also potently relaxes pulmonary vessels, and plays a pivotal role in the mediation of immune mechanisms^35^. VIPR2 and ADCYAP1R1, two receptors respectively for VIP and pituitary adenylate cyclase-activating polypeptide (PACAP, encoded by *Adcyap1*)^36^, are also shown to be down-regulated in the uterus of PD syndrome rats in this study. Similar to VIP, PACAP is a potent vasodilator. The vasodilating effect of VIP/PACAP is by way of smooth muscle cell receptors and activation of adenylate cyclase^37^. Therefore, the down-regulation of *Vip*, *Vipr2* and *Adcyap1r1*, clustered in the same module in the network, may altogether decrease cAMP and cyclase activities, resulting in the attenuation of USMC relaxation.

In the PPI network, *Vip*, along with *Vipr2* and *Adcyap1r1*, is connected to *Fos/Jun* group via a series of proteins that include G proteins/GTP binding proteins (guanine nucleotide binding protein (G protein), beta 1 and G protein subunit alpha 11, 14, 15 & q shown as *Gnb1*, *Gna11*, *Gna14*, *Gna15* and *Gnaq*), G-protein coupled receptor (arginine vasopressin receptor 1A shown as *Avpr1a*)^38^, RGS protein (regulator of G protein signaling 2 shown as *Rgs2*) that control signaling through G-protein coupled receptors^39^, and the downstream effectors of G protein (1-phosphatidylinositol 4,5-bisphosphate phosphodiesterase beta-3 & 4 shown as *Plcb3* and *Plcb4*)^40^. Noticeably, members of classical PI3K/Akt pathway links the G protein and its related proteins to *Fos/Jun* group. The PI3K (PIK3CD) can be activated by G protein-coupled receptors^41^. Through such connections, the up- and down-regulated genes co-work to participate in smooth muscle contraction. Interestingly, the interactions of these genes extend from nucleus to plasma membrane/extracellular position through cytosol. For future drug screen to relieve PD, the protein subcellular localization potentially provides targeting sites for special drugs. For instance, drugs increasing VIP may effect extracellularly while drugs decrease FOS/JUN group may be required to enter into nucleus.

Pseudoephedrine, a drug often used for nasal decongestant and bronchodilator, interacts with both FOS and JUN in the network. It has been reported that pseudoephedrine is able to activate *c-Fos* expression in rat nucleus accumbens and striatum^42^. However, there is also data showing that pseudoephedrine is capable of inhibiting T-cell activation by targeting AP-1 signaling pathways^43^. Currently, to our knowledge, little is clear about how pseudoephedrine effects on USMCs. Therefore, uncovering relations between pseudoephedrine and USMC functions are needed for further investigations.

FOS and JUN are two oncoproteins^44^. Drugs of arsenic trioxide, vinblastine and LGD-1550, interacting with JUN, along with nadroparin that interacts with FOS, all have anti-cancer activities^45–48^. Irbesartan, also interacting with JUN, has the anti-hypertensive effect^49^. These drugs may be potential to be used for PD treatment. In addition, *Calca*, encoding calcitonin gene-related peptide, is proven to be a vasodilator that promotes VSMC relaxation^50^. It interacts with olcegepant and MK-0974, which function as anti-migraine drugs^51^. This suggests that the two drugs may have the risk to enhance PD when used for remedying migraine.

## Conclusion

Our data, for the first time, analyze the mRNA expression profile of uterus from PD rats to construct PPI network. From the network, it can be seen that the up-regulated *Fos/Jun* group and the down-regulated *Vip* group are functionally linked. Through *Fos*/*Jun - Vip* connections, the roles of calcium ion, SMAD proteins and RNA polymerase II, as well as muscle stretch response, are associated with cAMP and cyclase activities. This is an elucidation of molecular regulations of PD in USMCs. Furthermore, the data provide the insight into finding new drug targets for PD relief. Therefore, in our next studies, drugs potential to target the key nodes in the PPI network are to be screened. This will greatly help to explore new approaches to PD prevention and treatment.

## Materials and Methods

### DEGs in the uterus of PD syndrome rat

The DEGs in the uterus of PD syndrome rat were shown in our previously published paper^9^. In brief, 682 DEGs, including 267 up-regulated and 415 down-regulated genes, were identified from the total RNA samples of the rat uterus with PD syndrome induced by estradiol benzoate and oxytocin via an Illumina NextSeq 500, 2 × 150 approach. These up- and down- regulated genes were respectively enriched into a series of pathways that closely related to uterine smooth muscle contraction and PD by ClueGo v 2.1.6, a Cytoscape plugin software. The pathways were also shown in the published paper and the associated DEGs implicated in the pathways were displayed in Supplemental Table S2. These DEGs in the uterus of PD syndrome rat were applied to PPI network analysis.

### Analyzing tool

A multi-functional online software NetworkAnalyst (http://www.networkanalyst.ca/)^52–55^ updated on 2017.09.15 was applied to analyze the DEGs in the uterus of PD syndrome rat for constructing the visualized PPI network. In addition, the analysis of path and module explorations, as well as protein-drug interactions, were also achieved by using this software. The prediction for protein subcellular localization was performed via online software Wolf Psort (https://www.genscript.com/wolf-psort.html).

### Generation of PPI network

The 682 DEGs were displayed together in a list with Ensembl gene IDs, along with the expressions that were shown as Log_2_ (fold change). The STRING Interactome was selected as the PPI database that is with medium (400) - high (1000) confidence score^56^. The confidence score cutoff was set as 900 for the analysis. The seeds were mapped to the corresponding molecular interaction database and the subnetworks with at least 3 nodes are demonstrated. The degree of each node was calculated based on the number of its connections to other nodes. In the network, The color of a certain node indicates its expression while the area represents the degree of it. The nodes with highest degrees were identified and shown as top nodes.

### Path exploring between nodes

Path explorer function in the software was used for visualizing the linked nodes that are functionally connected in the generated PPI network. To highlight the paths of interest, the connections were extracted and redesigned for convenient understanding. The nodes implicated in significantly enriched pathways (The DEGs in the corresponding cascades were shown in Supplemental Table S2 based on our previously published paper) that are closely related to USMC contraction and PD regulation were specifically marked in the connections.

### Module exploring in the PPI network

The module explorer in the software was applied for identifying the tightly clustered subnetworks in which the members are likely to function collectively. This was achieved by the Walktrap Algorithm, a random walk based strategy, for module detection. The *p* value of the module, calculated by a Wilcoxon rank-sum test of the degrees of the internal (the edges within a module) and external (the edges connecting the nodes of a module with the rest of the graph) edges, was considered significant when less than 0.05. The modules of interest were highlighted by different colors in the whole network.

### Protein-drug interactions analysis

The nodes of interest, including the up-regulated *Fos*, *Egr1*, *Jun*, *Junb*, *Jund*, *Fosb*, *Atf3* and *Klf2*, as well as the down-regulated *Vip*, *Vipr2*, *Adcyap1r1* and *Calca*, were analyzed to disclose their interactions with drugs. The Ensembl IDs of rat were converted to that of human prior to the analysis for the software only supporting human data. The drug and drug target information, collected from the DrugBank database (Version 5.0) in the software, was applied to match the nodes of interest to generate the protein-drug interactions network.

### Prediction for protein subcellular localization

The Ensembl ID of each up- or down-regulated gene was converted to that of Uniprot followed by obtaining the protein fasta. The prediction for the subcellular localization sites of proteins was based on the amino acid sequences through WoLF PSORT software.

## Electronic supplementary material

Supplemental Figures

Supplemental Tables
