# Supplementary material for: The PPI network analysis of mRNA expression profile of uterus from primary dysmenorrheal rats
Source: Sci Rep. 2018 Jan 10;8:351. doi: 10.1038/s41598-017-18748-2 (PMC5762641; doi:10.1038/s41598-017-18748-2)
Supplement: Supplementary file 1 — Supplemental Figures [file 41598_2017_18748_MOESM1_ESM.pdf]

The PPI network analysis of mRNA expression profile of uterus from primary dysmenorrheal rats

Pei Fan[1,*]: apisfp@126.com

Qiao-Hui Lin[1,*]: stacy0813@126.com

Ying Guo[1]: 13223080261@163.com

Lan-Ling Zhao[1]: 529712275@qq.com

He Ning[1]: 1470995684@qq.com

Meng-Ying Liu[1]: 15555883391@163.com

Dong-Qing Wei[2,3,#]: dqwei@sjtu.edu.cn

* These authors contribute equally to this work.

[#] Corresponding author

1. College of Biological Engineering, Henan University of Technology, Zhengzhou, China, 450001;

2. College of Chemical Engineering and Environment, Henan University of Technology, Zhengzhou, China, 450001;

3. College of Life Science and Biotechnology and State Key Laboratory of Microbial Metabolism, Shanghai Jiao Tong University, Shanghai, China, 200240

Supplemental Figures

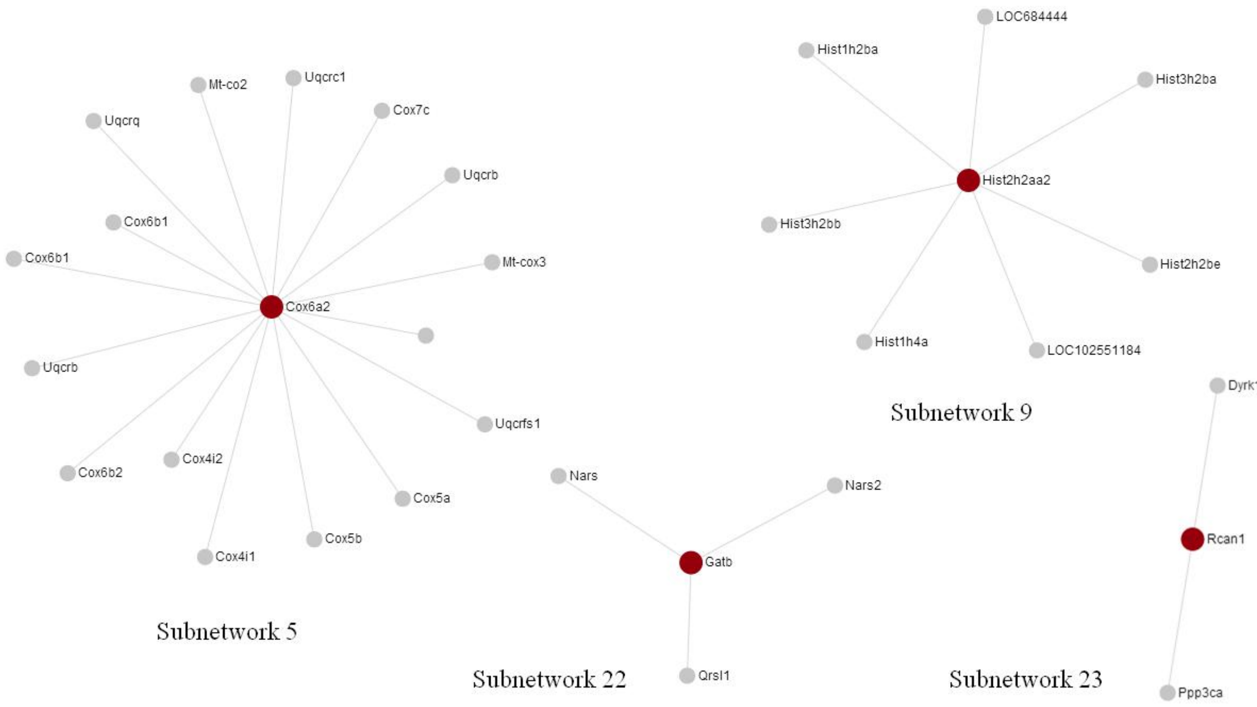

Supplemental Fig. S1 Subnetwork 5, 9, 22 and 23 of the PPI network. Nodes in red color are up-regulated in the uterus of PD syndrome rats. The grade of color and the area of node represent the expression level and the degree, respectively.

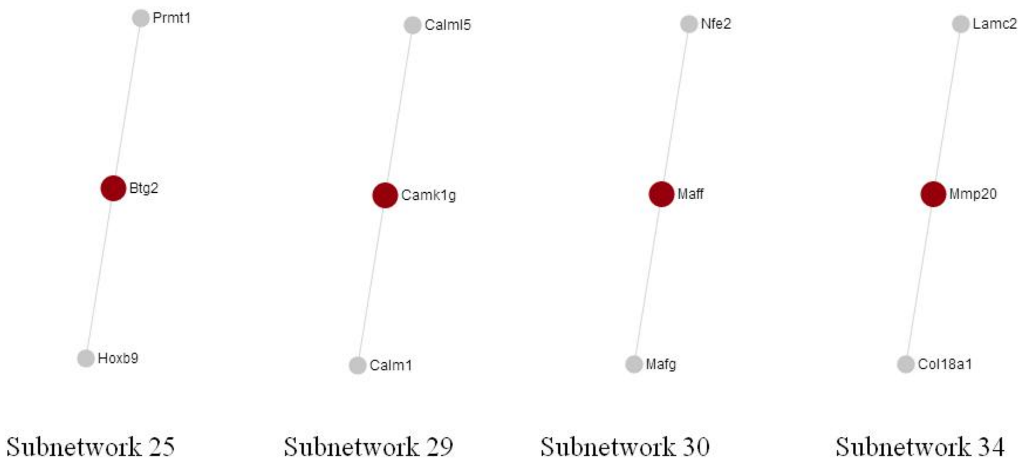

Supplemental Fig. S2 Subnetwork 25, 29, 30 and 34 of the PPI network. Red color symbolizes the up-regulated nodes. The color grade and node area represent the expression level and the degree, respectively.

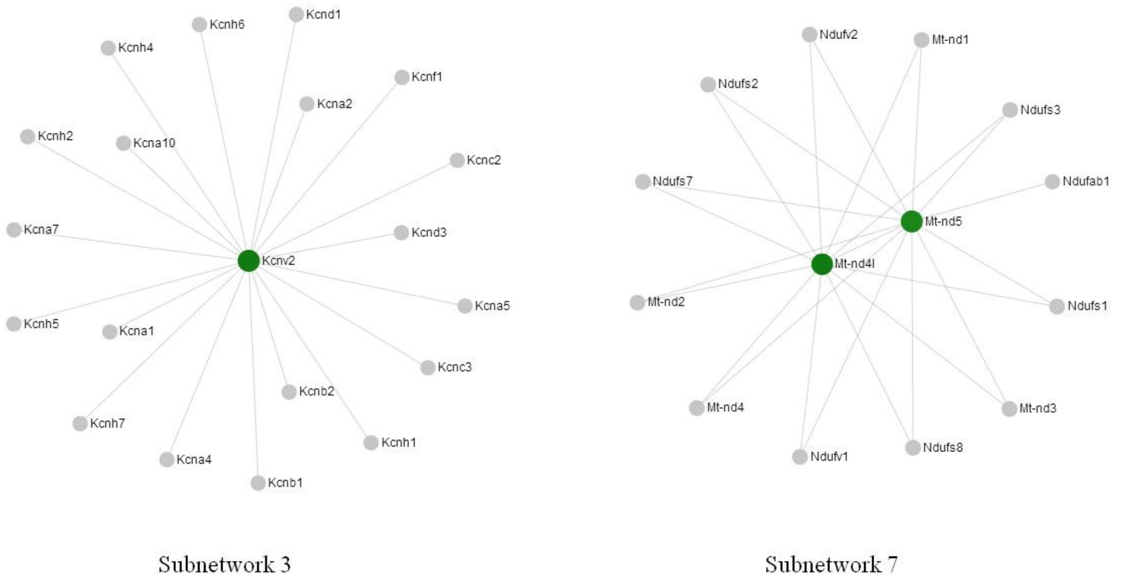

Supplemental Fig. S3 Subnetwork 3 and 7 of the PPI network. Green nodes are down-regulated in the uterus of PD syndrome rats. The expression level and the degree of the node are respectively represented by the grade of color and the area of node.

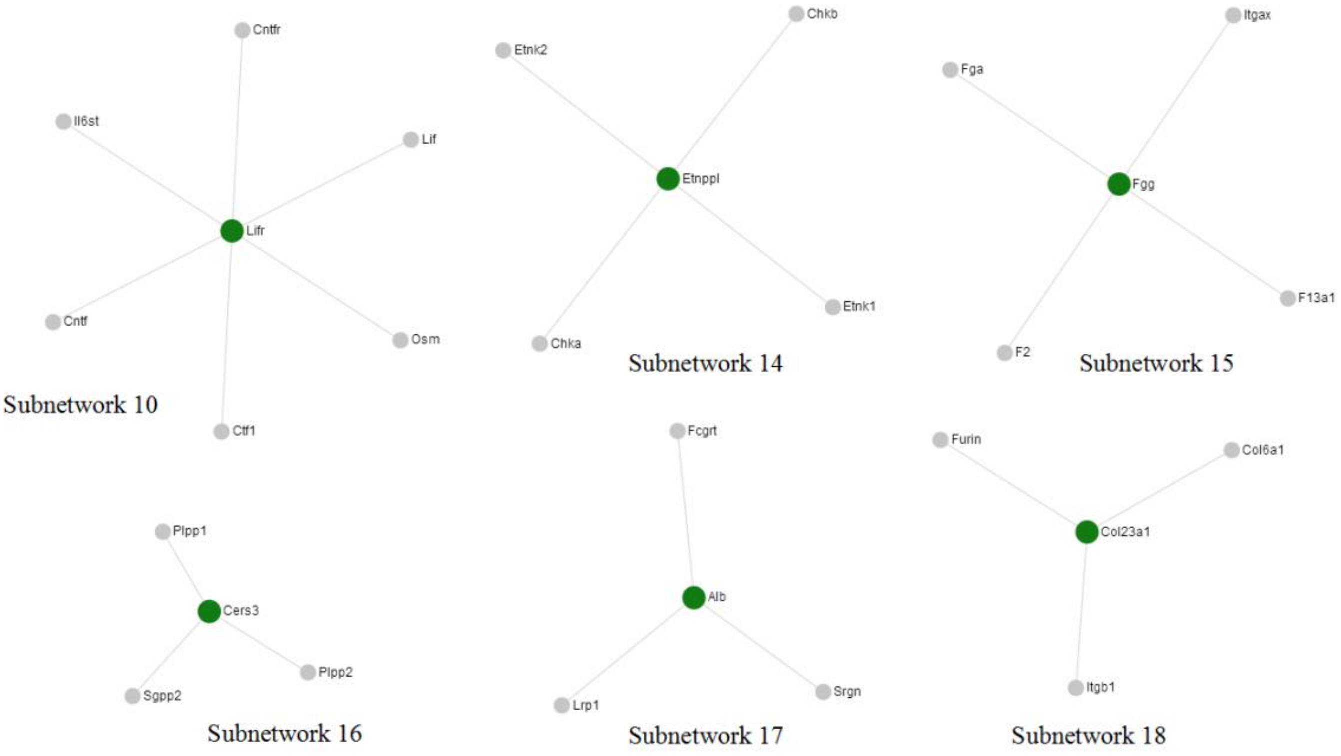

Supplemental Fig. S4 Subnetwork 10, 14, 15, 16, 17 and 18 of the PPI network. Nodes with green color indicate they are down-regulated. The grade of color and the area of node represent the expression level and the degree, respectively.

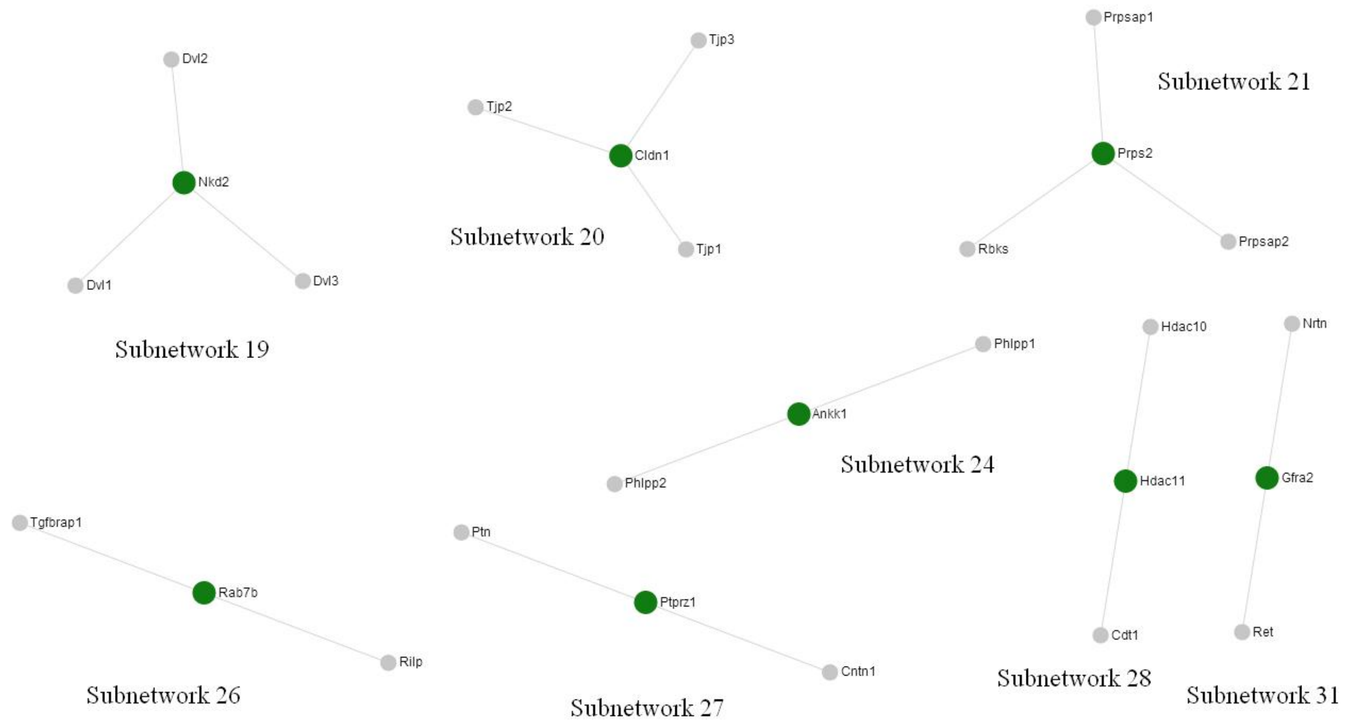

Supplemental Fig. S5 Subnetwork 19, 20, 21, 24, 26, 27, 28 and 31 of the PPI network. The down-regulated nodes are shown in green color. The grade of color represents its expression level while the area of node indicates and the degree.

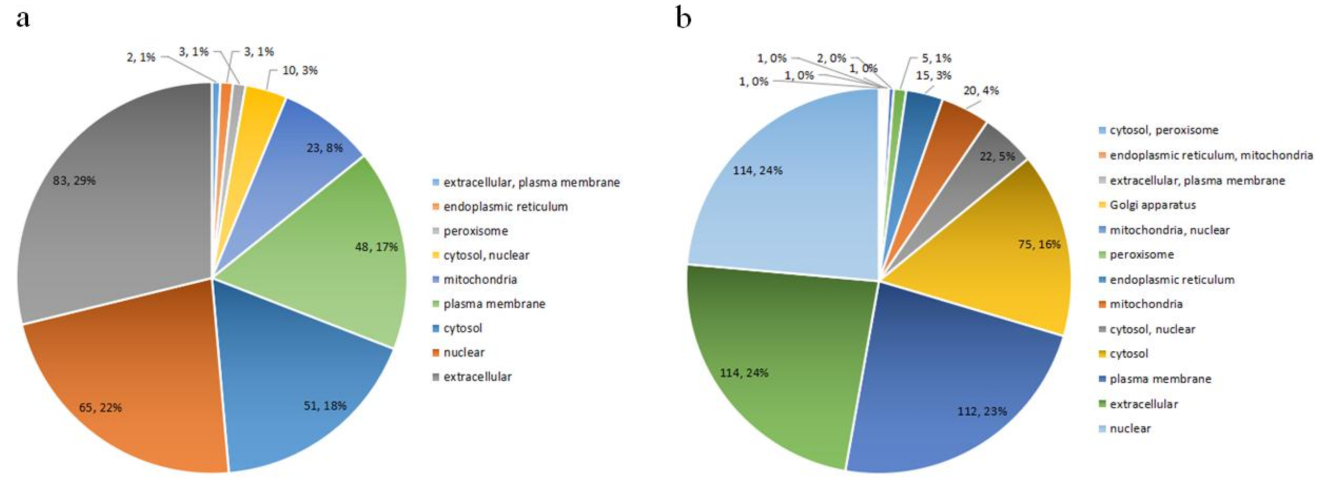

Supplemental Fig. S6 The protein subcellular localization for the differentially expressed mRNAs. (a) represents the categories and percentages for the locations of proteins of the up-regulated mRNAs while **(b)** indicates that of the down-regulated ones.
